# Immunometabolic Biomarkers in Colorectal Cancer: An Exploratory Diagnostic Evaluation of PBEF/NAMPT, Chemerin, and Osteopontin

**DOI:** 10.3390/ijms27188077

**Published:** 2026-09-11

**Authors:** Orest Szczygielski, Emilia Dąbrowska, Kamil Safiejko, Marcin Juchimiuk, Karolina Orywal, Monika Zajkowska

**Affiliations:** 1 Clinic of Paediatric Surgery, Institute of Mother and Child, Kasprzaka Str. 17a, 01-211 Warsaw, Poland; 2 General Hospital in Wysokie Mazowieckie, Szpitalna Str. 5, 18-200 Wysokie Mazowieckie, Poland; 3 Department of Oncological Surgery with Specialized Cancer Treatment Units, Maria Sklodowska-Curie Oncology Center, 15-027 Bialystok, Poland; kamil.safiejko@gmail.com (K.S.);; 4 Department of Biochemical Diagnostics, Medical University of Bialystok, 15-269 Bialystok, Poland; 5 Department of Neurodegeneration Diagnostics, Medical University of Bialystok, 15-269 Bialystok, Poland; monika.zajkowska@umb.edu.pl

**Keywords:** PBEF, NAMPT, chemerin, OPN, novel biomarkers, biomarker discovery, diagnostic panel

## Abstract

Immunometabolic dysregulation plays a pivotal role in colorectal cancer (CRC) development and progression. Among the molecules linking inflammation, metabolism, and tumorigenesis, pre-B-cell colony-enhancing factor/nicotinamide phosphoribosyltransferase (PBEF/NAMPT), chemerin, and osteopontin (OPN) have emerged as promising circulating biomarkers. This single-center study evaluated their diagnostic performance in patients with colorectal cancer and compared their utility with established tumor markers. Serum concentrations of PBEF/NAMPT, chemerin, and OPN were determined using a magnetic bead-based multiplex immunoassay (Luminex technology), whereas carcinoembryonic antigen (CEA) and carbohydrate antigen 19-9 (CA19-9) were measured by chemiluminescent microparticle immunoassay (CMIA). The study included 76 patients with colorectal cancer and 38 healthy, colonoscopy-negative controls. Differences between groups were assessed using the Mann–Whitney U, Kruskal–Wallis and post hoc tests, and diagnostic performance was evaluated by receiver operating characteristic (ROC) curve analysis. Serum concentrations of PBEF/NAMPT and OPN were significantly higher in patients with CRC than in healthy controls, whereas chemerin did not differ significantly between groups. Among the investigated immunometabolic biomarkers, OPN demonstrated the highest diagnostic accuracy (AUC = 0.747), followed by PBEF/NAMPT (AUC = 0.624), whereas chemerin showed no significant diagnostic value (AUC = 0.559). Combining immunometabolic biomarkers with CEA yielded numerically higher AUC values than CEA alone, with the OPN + CEA panel reaching the highest diagnostic accuracy (AUC = 0.910), followed by the PBEF/NAMPT + CEA combination (AUC = 0.875); however, these increases did not reach statistical significance when formally compared with CEA alone. Among the evaluated immunometabolic biomarkers, OPN demonstrated the greatest diagnostic potential for colorectal cancer, and its combination with CEA warrants further investigation, although these findings should be considered exploratory given the limited sample size and the absence of external validation. Larger, multicenter studies using clinically relevant comparator groups are needed to establish the clinical utility of such multimarker strategies for the non-invasive diagnosis of CRC.

## 1. Introduction

Colorectal cancer (CRC) remains one of the most significant oncological challenges worldwide. It is the third most frequently diagnosed malignancy, with more than 1.9 million new cases and approximately 935,000 deaths reported annually [1]. Although advances in screening programs and therapeutic approaches have improved patient outcomes, CRC continues to be associated with high mortality, particularly among patients diagnosed at advanced stages. Tumor progression is not determined solely by genetic alterations but also involves extensive metabolic remodeling and persistent inflammatory activation. Therefore, increasing attention has been directed toward biomarkers reflecting the complex interactions between metabolism, inflammation, and cancer development [2].

Serum biomarkers are widely used in clinical oncology because blood collection is minimally invasive, relatively inexpensive, and compatible with routine diagnostic procedures [3]. In CRC, carcinoembryonic antigen (CEA) and carbohydrate antigen 19-9 (CA19-9) remain the most commonly applied circulating markers, mainly for disease monitoring and evaluation of treatment response. However, their diagnostic performance is limited, particularly when used as single markers. Reported sensitivity values range from 65% to 74% for CEA and from 26% to 48% for CA19-9 [4,5]. Although CA19-9 demonstrates lower sensitivity, its association with CEA indicates that combined biomarker strategies may provide additional diagnostic information [6,7]. These limitations highlight the need to identify novel circulating biomarkers that could complement currently available diagnostic tools.

The clinical course of CRC depends on multiple factors, including tumor stage, biological characteristics, and host response. The TNM (Tumor-Node-Metastasis) classification remains the most important system for assessing disease advancement and guiding therapeutic decisions; however, patients with the same stage may present different clinical outcomes, suggesting the involvement of additional molecular mechanisms affecting tumor behavior. Biomarkers associated with inflammatory and metabolic alterations may therefore provide valuable information regarding tumor biology and disease progression. Recent evidence indicates that cancer-associated metabolic changes are closely linked with immune regulation. This concept, referred to as immunometabolism, describes the interaction between metabolic pathways and immune responses that influence tumor initiation, growth, and the composition of the tumor microenvironment [8,9,10]. In CRC, chronic inflammation and metabolic disturbances contribute to carcinogenesis and may promote tumor progression through altered cytokine signaling, immune cell activation, and changes in cellular metabolism. Consequently, circulating molecules involved in these processes have emerged as potential candidates for diagnostic and prognostic evaluation. Among immunometabolic molecules, pre-B-cell colony-enhancing factor/nicotinamide phosphoribosyltransferase (PBEF/NAMPT), chemerin, and osteopontin (OPN) have attracted particular interest due to their involvement in inflammatory regulation and metabolic homeostasis. Nevertheless, the clinical significance of circulating concentrations of these biomarkers in CRC remains unclear, and their diagnostic performance in comparison with established tumor markers requires further investigation [11].

PBEF/NAMPT is an enzyme involved in the salvage pathway of NAD biosynthesis, converting nicotinamide into nicotinamide mononucleotide (NMN). Through regulation of intracellular NAD availability, PBEF/NAMPT influences several cellular processes, including energy metabolism, DNA repair, transcriptional regulation, and cell-cycle control. Increased PBEF/NAMPT activity has been described in different malignancies and may contribute to tumor cell survival by supporting metabolic adaptation and proliferation [12].

Chemerin is an adipokine involved in both metabolic regulation and inflammatory signaling. Increased circulating chemerin levels have been associated with inflammatory mediators such as tumor necrosis factor-alpha (TNF-α), interleukin-6 (IL-6), and C-reactive protein (CRP), indicating its involvement in systemic inflammatory responses [13,14]. In the context of cancer, chemerin may influence immune cell recruitment and interactions within the tumor microenvironment, although its diagnostic relevance in CRC remains to be fully established.

OPN is a secreted phosphoprotein involved in cell adhesion, migration, and survival. Due to its cytokine-like properties, it regulates inflammatory responses and immune cell activity. Previous studies have demonstrated that OPN may contribute to tumor progression by promoting invasion, angiogenesis, and metastatic potential, making it a promising candidate for cancer biomarker research, including CRC [15].

Taken together, PBEF/NAMPT, chemerin, and OPN represent biologically relevant molecules linking metabolic alterations with chronic inflammation, two processes strongly involved in colorectal carcinogenesis and progression [16]. However, available data regarding their diagnostic usefulness in CRC are still limited, particularly when evaluated alongside routinely used tumor markers and across different stages of disease. Investigation of these biomarkers may provide additional insight into their potential role in CRC detection and biological characterization. Therefore, the aim of this study was to evaluate serum concentrations and diagnostic performance of PBEF/NAMPT, chemerin, and OPN in patients with colorectal cancer compared with healthy controls and to determine their potential usefulness as immunometabolic biomarkers in comparison with established serum tumor markers, including CEA and CA19-9.

## 2. Results

Table 1 presents the serum concentrations of PBEF/NAMPT, OPN, chemerin, CEA, and CA19-9 in patients with colorectal cancer and healthy controls. Comparison of the two groups using the Mann–Whitney U test demonstrated significantly elevated serum levels of PBEF/NAMPT, OPN, CEA, and CA19-9 in the CRC group. In contrast, although chemerin concentrations tended to be higher in patients with colorectal cancer, the difference did not reach statistical significance.

Subsequently, subgroup analyses were performed using the Mann–Whitney U test to determine whether biomarker concentrations differed according to tumor location (colon vs. rectum) or subsequent administration of neoadjuvant treatment (radiotherapy and/or chemotherapy) before surgical intervention. No significant differences were observed between patients who received preoperative oncological treatment and those who underwent surgery without prior therapy. However, OPN concentrations were significantly higher in patients with rectal cancer than in those with colon cancer (*p* = 0.032; colon: median 61,158.40 pg/mL, range 3537.98–368,948.80 pg/mL; rectum: median 86,150.58 pg/mL, range 3670.57–663,582.50 pg/mL). To further investigate the relationship between biomarker levels and disease progression, patients were stratified according to TNM stage into early-stage (stages I–II) and advanced-stage disease (stages III–IV), as well as into all four TNM stages. Differences among these groups were evaluated using the Kruskal–Wallis test followed by post hoc multiple-comparison analysis. Statistically significant differences were identified for all analyzed biomarkers across disease stages (Table 2 and Table 3).

Post hoc analysis (Table 2) revealed that serum PBEF/NAMPT concentrations were significantly higher in patients with early-stage CRC than in healthy controls (*p* = 0.011), whereas no significant differences were observed between controls and advanced-stage disease and between advancement groups. OPN concentrations were significantly elevated in both early- and advanced-stage CRC compared with controls (*p* < 0.001 and *p* = 0.002, respectively), although no significant difference was detected between the two cancer groups. In contrast, chemerin levels distinguished patients with early-stage CRC from healthy individuals (*p* = 0.008) and further decreased with the advancement of the disease, resulting in a significant difference between early- and advanced-stage CRC (*p* < 0.001). Similarly, CEA concentrations were significantly higher in both CRC subgroups than in controls and additionally discriminated between early and advanced disease (*p* < 0.001). For CA19-9, a significant increase was observed only in patients with advanced-stage CRC compared with healthy controls (*p* = 0.011).

**Figure 1:**
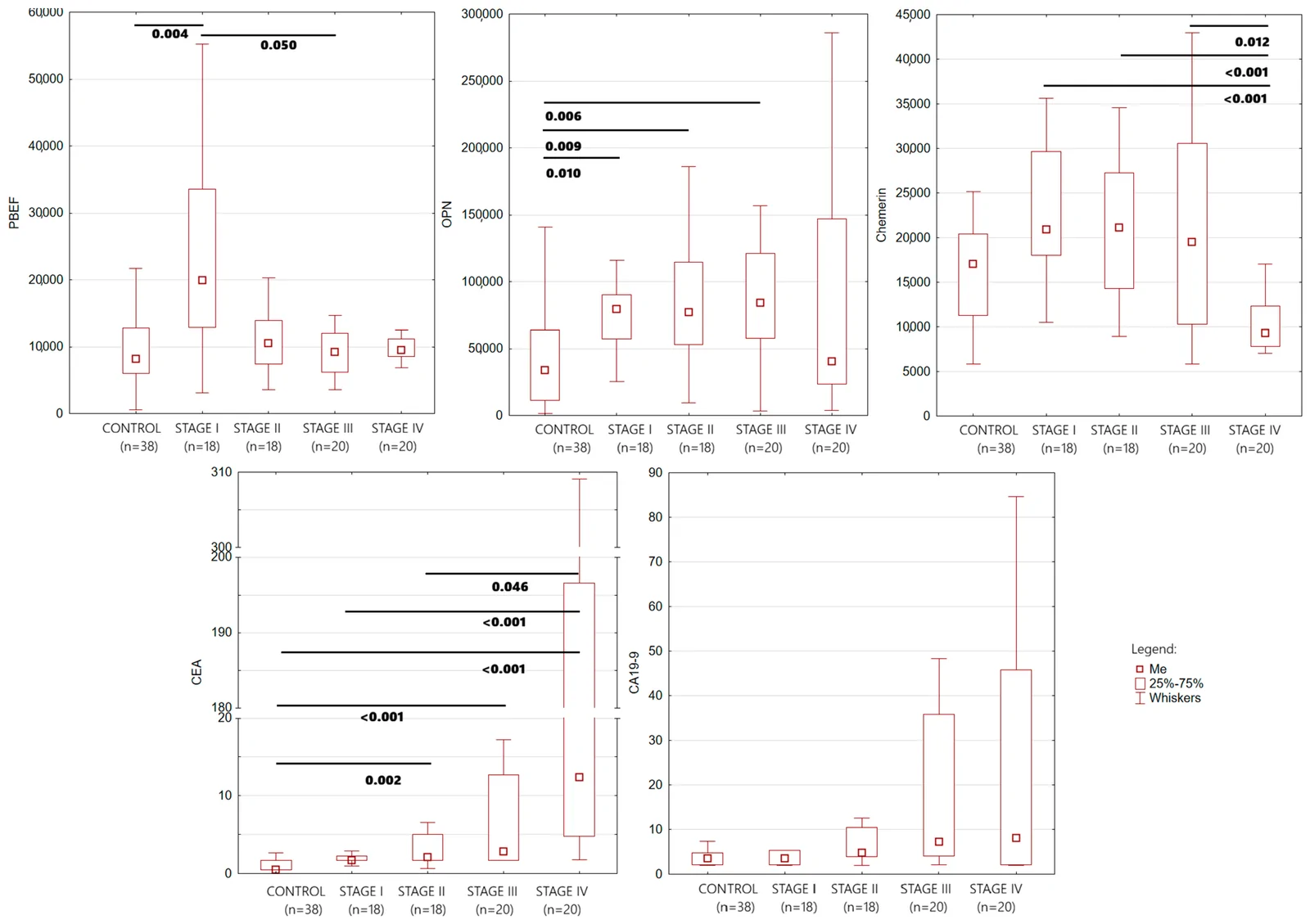
Distribution of serum concentrations of PBEF/NAMPT, chemerin, OPN, CEA, and CA19-9 according to TNM stage in patients with colorectal cancer and healthy controls. Outliers and extreme values were excluded.

To further characterize biomarker expression across individual TNM stages, CRC patients were subsequently divided into stages I–IV. Figure 1 presents box-and-whisker plots illustrating the distribution of serum concentrations of PBEF/NAMPT, chemerin, osteopontin (OPN), CEA, and CA19-9 in healthy controls and patients with colorectal cancer stratified according to TNM stage. Interestingly, the distribution of PBEF/NAMPT concentrations differed across disease stages, with the highest median values observed in patients with stage I CRC and lower median concentrations in subsequent stages. Chemerin concentrations were comparable between the control group and stages I–III but were lower in patients with stage IV disease. OPN concentrations were elevated in all CRC stages compared with healthy controls. CEA demonstrated a progressive increase with advancing disease stage, with the highest concentrations and the greatest variability recorded in stage IV CRC. A similar trend was observed for CA19-9, where the widest distribution and the highest extreme values occurred in patients with advanced disease.

The box-and-whisker plots suggested stage-dependent differences in biomarker concentrations; therefore, a more detailed comparison across individual TNM stages was performed using the Kruskal–Wallis test followed by post hoc multiple-comparison analysis (Table 3). The median concentrations of PBEF/NAMPT, OPN, Chemerin, CEA, and CA19-9 according to CRC stage are presented in Supplementary Table S1.

Significant overall differences among the groups were observed for all biomarkers. PBEF/NAMPT concentrations were significantly elevated in stage I compared with the control group (*p* = 0.004), while only a borderline difference was found between stages I and III (*p* = 0.050). OPN levels were significantly higher in stages I, II, and III than in healthy controls, suggesting that this biomarker is altered already in the early phases of disease. CEA demonstrated a progressive increase with tumor advancement, with significant differences between controls and stages II, III, and IV, as well as between stage IV and both stages I and II. Interestingly, no differences were observed between controls and stage I. Chemerin concentrations were primarily associated with advanced disease, as significant differences were observed only between stage IV and stages I, II and III. In contrast, CA19-9 showed only limited discriminatory ability, with significant overall differences in the Kruskal–Wallis analysis but no statistically significant pairwise comparisons after post hoc testing.

To formally test for a trend across TNM stages, the Jonckheere–Terpstra test was applied. A statistically significant decreasing trend was confirmed for PBEF/NAMPT across TNM stages I–IV (JT = 826.5, *p* = 0.0089; median concentrations: 19,956.0, 10,516.0, 9211.5, and 9538.7, respectively). In contrast, no significant monotonic trend was detected for OPN (JT = 1014, *p* = 0.737); OPN concentrations were numerically higher in stages I–III (medians 79,594.7, 77,066.1, and 84,184.1, respectively) but markedly lower in stage IV (median 40,398.6), indicating a non-linear pattern rather than a consistent trend with increasing tumor stage.

Table 4 summarizes the diagnostic performance of the investigated biomarkers, including the area under the ROC curve (AUC), sensitivity (SE), specificity (SP), accuracy (ACC), positive predictive value (PPV), and negative predictive value (NPV). Among the novel biomarkers, OPN demonstrated the highest diagnostic performance, with an AUC of 0.747, sensitivity of 68.4%, specificity of 71.1%, and diagnostic accuracy of 69.3%. Although PBEF/NAMPT exhibited a lower AUC (0.624), it achieved the highest sensitivity among the newly investigated biomarkers (69.7%). In contrast, chemerin did not show significant individual diagnostic value. Among the routinely used tumor markers, CEA demonstrated the best overall diagnostic performance, yielding the highest AUC (0.864), sensitivity (92.1%), accuracy (85.1%), NPV (81.8%), and a high PPV (86.4%). CA19-9 showed considerably lower diagnostic utility, with an AUC of 0.632, sensitivity of 51.3%, specificity of 78.9%, and accuracy of 60.5%.

To evaluate whether the novel biomarkers could improve the diagnostic performance of CEA, logistic regression models combining CEA with PBEF/NAMPT or OPN were constructed. Both combined models showed numerically higher AUC values than the individual biomarkers. The combination of PBEF/NAMPT and CEA yielded an AUC of 0.875, with a specificity of 89.5% and a PPV of 93.1%. The numerically highest AUC among all tested models was achieved by the OPN + CEA combination (AUC = 0.910), together with a sensitivity of 82.9%, specificity of 89.5%, accuracy of 85.1%, PPV of 94.0%, and NPV of 72.3%. Bootstrap-based optimism correction (500 replicates) indicated minimal overfitting for both combined models, with an estimated optimism of 0.006 for both PBEF/NAMPT + CEA and OPN + CEA, yielding optimism-corrected AUC values of 0.869 and 0.904, respectively (compared with apparent AUC values of 0.875 and 0.910). However, when the AUC of each combined model was formally compared with the AUC of CEA alone using a paired DeLong test, the numerical increase in AUC did not reach statistical significance for either model (OPN + CEA: ΔAUC = 0.046, *p* = 0.095; PBEF/NAMPT + CEA: ΔAUC = 0.011, *p* = 0.667), a result confirmed by bootstrap resampling (*p* = 0.093 and *p* = 0.665, respectively).

**Figure 2:**
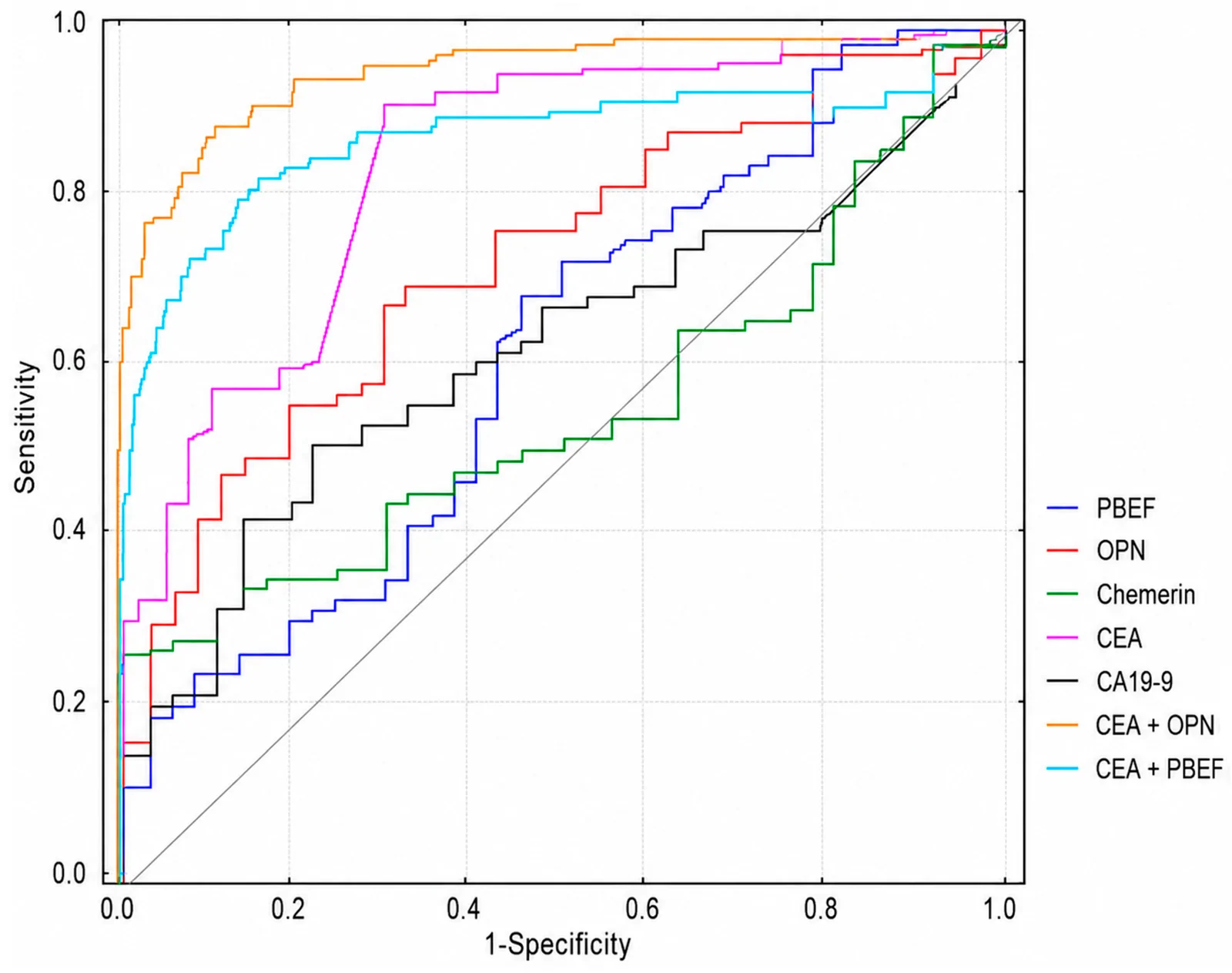
Receiver operating characteristics for all parameters.

The graphical representation of all ROC analyses is provided in Figure 2. Among the individual biomarkers, AUCs for PBEF/NAMPT, OPN, CEA, and CA19-9 were significantly greater than 0.5 (*p* < 0.05 for all), whereas chemerin showed no significant individual diagnostic value (AUC = 0.559, 95% CI 0.454–0.665, *p* = 0.273). Because the CRC and control groups differed significantly in age (median 66.5 vs. 48.5 years, *p* < 0.001, Mann–Whitney U test) and sex distribution (65.8% vs. 42.1% male, *p* = 0.026, Fisher’s exact test), logistic regression models were additionally adjusted for age and sex. In these adjusted models, both PBEF/NAMPT (*p* = 0.023) and OPN (*p* = 0.002) remained independently associated with CRC status alongside CEA (*p* = 0.023 and *p* = 0.008, respectively) and age (*p* < 0.001 in both models), while sex was not an independent predictor (*p* = 0.43–0.56). The AUC of the age- and sex-adjusted combined models was 0.918 for PBEF/NAMPT + CEA and 0.938 for OPN + CEA, compared with an AUC of 0.907 for a model containing only CEA, age, and sex; this difference did not reach statistical significance for either biomarker (OPN: ΔAUC = 0.031, *p* = 0.094; PBEF/NAMPT: ΔAUC = 0.011, *p* = 0.366, paired DeLong test). In both unadjusted and age/sex-adjusted logistic regression models, PBEF/NAMPT and OPN remained statistically significant independent predictors of CRC status alongside CEA (*p* < 0.05 for all), while sex was not an independent predictor after adjustment for age and biomarker concentration. Regression coefficients, odds ratios (including per-1000 pg/mL increments for interpretability), and 95% confidence intervals for all models are provided in Supplementary Table S2.

Additional diagnostic performance of individual biomarkers and their combinations for discriminating only early-stage CRC (TNM stages I–II) from healthy controls is presented in Supplementary Table S3.

**Figure 3:**
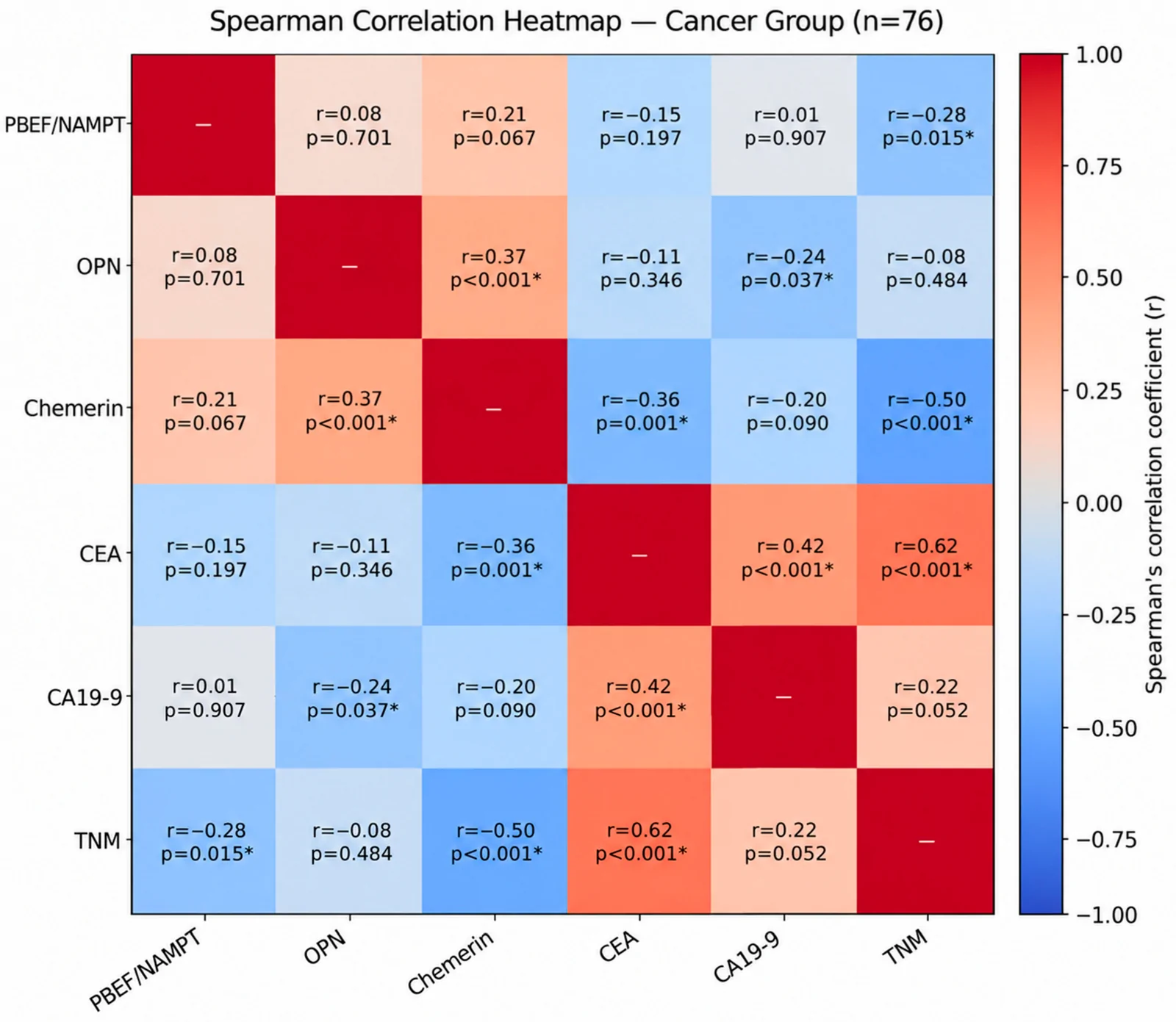
Spearman’s rank correlation coefficient for the tested variables (only CRC patients). TNM stage was coded as an ordinal variable (I–IV = 1–4); *—statistically significant correlations.

To complement the statistical analysis, Spearman’s rank correlation coefficient was calculated to assess the strength and direction of the monotonic relationship between variables. The obtained results are shown in Figure 3.

Spearman’s correlation analysis in the CRC group revealed that CEA exhibited the strongest positive correlation with TNM stage (r = 0.62, *p* < 0.001), indicating that serum CEA concentrations increased with colorectal cancer progression. CEA was also moderately positively correlated with CA19-9 (r = 0.42, *p* < 0.001) and moderately negatively correlated with chemerin (r = −0.36, *p* = 0.001). Chemerin also showed a moderate-to-strong negative correlation with disease stage (r = −0.50, *p* < 0.001). PBEF/NAMPT showed a weak negative correlation with disease stage (r = −0.28, raw *p* = 0.015), which did not remain significant after Bonferroni correction for multiple testing; this is consistent with the significant Jonckheere–Terpstra trend test for PBEF/NAMPT reported separately, which used a dedicated, targeted trend analysis rather than this exploratory correlation matrix. OPN showed a moderate positive correlation with chemerin (r = 0.37, *p* < 0.001); its negative correlation with CA19-9 (r = −0.24, raw *p* = 0.037) did not remain significant after Bonferroni correction. All correlations were assessed using Spearman’s rank coefficient in the CRC group only (*n* = 76), with Bonferroni correction applied across the 15 pairwise comparisons.

## 3. Discussion

Colorectal cancer is the third most common malignancy worldwide in terms of both incidence and mortality [17]. Chronic inflammation is considered an important prognostic factor in the course of this disease. In addition, accumulating evidence suggests that the inflammatory tumor microenvironment influences cancer initiation, progression, tumor growth, and metastasis. Numerous studies support the role of chronic inflammation in the development of colorectal cancer [18]. Inflammatory mediators are involved in the transformation of adenoma cells into adenocarcinoma cells in the colon [19]. However, despite these findings, the role of inflammatory biomarkers in colorectal carcinogenesis remains inconclusive and requires further investigation. Numerous studies have indicated that selected immunometabolic biomarkers may contribute to tumor development and modulate key processes involved in cancer progression, including angiogenesis, proliferation, and metastasis [20]. Examples of such molecules include PBEF/NAMPT, chemerin, and OPN, which have been shown to participate in tumor angiogenesis and are associated with the pathogenesis of several malignancies, including colorectal cancer (CRC). These proteins have been detected in both plasma and tumor tissues of cancer patients, suggesting their potential diagnostic value [21,22,23].

Our study demonstrated that serum PBEF/NAMPT and OPN concentrations were significantly higher in CRC patients than in healthy controls, similarly to the classical tumor biomarkers CEA and CA19-9. These findings are consistent with previous reports showing elevated serum PBEF/NAMPT and OPN levels in CRC patients compared with healthy individuals. In a total colonoscopy-based cross-sectional case–control study, Yagi et al. demonstrated that serum chemerin levels were significantly higher in male patients with colorectal adenoma than in the control group [24]. Overexpression of the PBEF/NAMPT gene and protein has also been demonstrated in CRC using a global mass spectrometry-based metabolomic approach [25]. Rectal cancer often shows a high-grade local inflammatory reaction at the tumor margin, whereas colon cancer tends to present with more pronounced systemic inflammation. The immune cells (like lymphocytes and macrophages) gather right around the tumor. OPN plays an important role in both acute and chronic inflammatory states—it mediates the migration of macrophages into the sites of inflammation, induces IL-12, IFN-γ production by macrophages. In our opinion, this may be one of the reasons for the higher OPN levels observed in rectal cancer compared with colon cancer. Moreover, the interaction between OPN and the cell surface receptor CD44 coordinates macrophage recruitment, motility, and activation during inflammation and tumor progression. Furthermore, macrophages cultured with CD44-positive colorectal cancer cells were able to produce higher levels of OPN. Since relatively few studies have investigated circulating concentrations of PBEF/NAMPT, chemerin, and OPN in colorectal cancer, we additionally focused on studies evaluating their tissue expression and gene expression. Mattos et al. demonstrated significantly higher expression levels of OPN splice variants in CRC samples than in healthy tissues [26]. Similarly, analyses of PBEF/NAMPT expression in adenoma and carcinoma tissues revealed markedly higher expression levels than in normal colorectal tissue. Furthermore, PBEF/NAMPT overexpression has been associated with poorer prognosis in CRC patients [27]. Previous immunohistochemical studies also demonstrated that high PBEF/NAMPT expression correlated positively with tumor invasion, advanced TNM stage, and shorter patient survival [28,29]. Taken together, these findings are consistent with our results showing elevated circulating PBEF/NAMPT and OPN concentrations in CRC patients. Although the biological role of these proteins has been investigated mainly at the tissue level, our findings suggest that their altered expression is also reflected in serum and may therefore have potential clinical utility as minimally invasive biomarkers.

In our analysis according to TNM stage, PBEF/NAMPT concentrations were significantly elevated in stage I compared with controls (*p* = 0.004), with no significant difference between controls and stages II, III, or IV. A Jonckheere–Terpstra trend test confirmed a significant overall decreasing trend across TNM stages I–IV (*p* = 0.0089), consistent with a peak in early disease followed by a gradual decline toward baseline values rather than a difference sustained at every subsequent stage. Reports regarding the relationship between circulating PBEF/NAMPT levels and TNM stage have previously been inconsistent; although several studies found significantly higher PBEF/NAMPT concentrations in CRC patients than in healthy controls, they did not demonstrate significant differences between PBEF/NAMPT levels and TNM stage, suggesting that PBEF/NAMPT may be a marker of tumor malignancy rather than disease stage [30,31]. Our findings, supported by a formal ordered trend test, provide evidence for a stage-related pattern in this cohort, although this apparent discrepancy with prior reports may reflect differences in sample size, patient selection, or analytical methods between studies. The lack of a significant Bonferroni-corrected Spearman correlation between PBEF/NAMPT and TNM stage (reported separately), despite this significant Jonckheere–Terpstra trend, likely reflects the fact that the trend test is specifically designed to detect ordered, non-monotonic patterns such as the early peak observed here, whereas a single correlation coefficient assumes a more uniform relationship across the full range.

OPN and chemerin displayed a similar, non-monotonic pattern: both were maintained at a relatively stable, elevated level across stages I–III, with a marked decrease in stage IV (post hoc comparisons: stage III vs. IV, *p* = 0.006 for OPN and *p* = 0.012 for chemerin; stage I vs. IV, *p* < 0.001 for both). Accordingly, a Jonckheere–Terpstra test did not support a monotonic trend for OPN across stages (*p* = 0.737), indicating that this pattern reflects a plateau in early-to-locally advanced disease followed by a decline in metastatic disease, rather than a consistent linear trend. Notably, OPN concentrations differed significantly from controls in stages I, II, and III (*p* = 0.010, 0.009, and 0.006, respectively) but not in stage IV (*p* = 0.319), suggesting that this biomarker may lose diagnostic value once distant metastases have developed. In contrast, chemerin did not differ significantly from controls at any individual stage, consistent with its limited overall diagnostic performance (AUC = 0.559); nevertheless, its concentration still declined significantly in stage IV relative to earlier stages, indicating that chemerin dynamics may still reflect disease progression even though they do not discriminate CRC patients from healthy individuals. In some solid tumors, such as colorectal cancer, the concentration of chemerin in the cancer tissue may decrease with increasing tumor stage. This may be related to the fact that cancer cells silence the action of certain proteins to avoid an immune response. Cancer cells can evade the immune system by downregulating or losing the expression of the proteins recognized by the immune cells as antigens. Chemerin acts as a chemoattractant that can recruit natural killer (NK) cells and other immune defenses to tumor microenvironments in various cancers. To survive and thrive, malignant cells must evade or subvert antitumor immune responses. Malignant cells can down-regulate NK cells, and thereby the expression of proteins that recruit these cells to the tumor microenvironment.

The mechanisms underlying the decline in OPN and chemerin concentrations in stage IV disease remain unclear. One possible explanation is altered protein synthesis, secretion, or clearance associated with the systemic burden of metastatic disease; however, this remains speculative, and mechanistic or longitudinal studies would be needed to clarify these dynamics. The development of new diagnostic methods enabling early detection of colorectal cancer remains of great importance, as it may significantly reduce disease-related mortality. Non-invasive biomarker-based diagnostic approaches represent an attractive strategy that could shorten the time to diagnosis, particularly if biomarkers such as OPN retain diagnostic value in the early and locally advanced stages where it is most needed. However, the mechanisms underlying these changes remain unclear and require further investigation.

To the best of our knowledge, this is the first study evaluating the diagnostic usefulness of PBEF/NAMPT, OPN, and chemerin together with the established CRC biomarkers CEA and CA19-9 within a single diagnostic panel. The diagnostic sensitivity of PBEF/NAMPT, and OPN increased when they were combined with CEA (chemerin was excluded from combined biomarker panels because it did not demonstrate significant individual diagnostic value). The highest diagnostic sensitivity was achieved for the combined assessment of OPN and CEA. Although CEA demonstrated the highest diagnostic performance among the individual biomarkers, combining it with OPN showed higher AUC values. This suggests that OPN may provide complementary biological information rather than simply reflecting the same pathological processes as CEA. Such combined biomarker panels may therefore represent a more effective diagnostic approach than the assessment of a single circulating marker. These findings should be considered exploratory and require confirmation in independent cohorts, since the numerically highest AUC observed for the OPN + CEA combination did not reach statistical significance compared with CEA alone—likely reflecting the limited sample size and consequently limited statistical power to detect AUC differences of this magnitude. Larger, adequately powered studies are needed to confirm whether the addition of OPN meaningfully improves the diagnostic performance of CEA. Based on the analysis of elevated biomarker concentrations, we demonstrated that combining these proteins with the classical tumor marker CEA may be more useful for CRC diagnosis than measuring individual biomarkers alone. In a prospective study by Chen et al. evaluating the role of PBEF/NAMPT in colonic polyps, the AUC of extracellular NAMPT for predicting the prominent malignant potential (PMP) of colonic polyps was 0.766 (sensitivity 72.34%; specificity 78.93%), whereas the AUC for CEA was only 0.534 [32].

When comparing our findings with the existing literature, the relationship between circulating chemerin concentrations and CRC progression remains inconsistent. Waniczek et al. reported that both serum and salivary chemerin concentrations may serve as valuable biomarkers for the early diagnosis of CRC, with higher levels distinguishing early-stage patients from healthy controls [33]. In our cohort, however, chemerin did not significantly differentiate any individual TNM stage from controls, and its overall diagnostic performance was poor (AUC = 0.559); instead, we observed a distinct decline specifically in stage IV compared with earlier stages. Another study found that serum chemerin concentrations reflected tumor stage according to the TNM classification, with higher levels associated with more advanced disease and significantly higher concentrations in CRC patients than in healthy controls [22]—a direction opposite to the decrease we observed in stage IV. These discrepancies may be explained by differences in study design, patient selection, analytical methods, and the relatively small number of patients per stage in both our study and those cited, which limits the reliability of stage-specific comparisons across studies. Further studies with larger, well-stratified cohorts are needed to clarify the relationship between circulating immunometabolic biomarkers and colorectal cancer progression. This study has several limitations. First, it was conducted at a single center and included a relatively limited number of participants, particularly after stratification according to TNM stage, which may have reduced the statistical power of some subgroup analyses. Second, serum biomarker concentrations were assessed at a single time point before treatment, preventing evaluation of their changes during therapy or follow-up. Third, the control group consisted of extensively screened healthy volunteers with a negative colonoscopy, normal laboratory parameters, and no obesity, inflammatory, or gastrointestinal disease. While this provided a well-characterized comparator, none of the three novel biomarkers investigated is tumour-specific, and all are influenced by systemic inflammation and metabolic status independently of malignancy. The diagnostic performance reported here is therefore likely overestimated due to spectrum bias, relative to what would be observed against a more clinically relevant comparator group, such as patients with colorectal polyps, adenomas, inflammatory bowel disease, or diverticular disease. The CRC and control groups also differed significantly in age and sex distribution, both of which are known to influence NAMPT and chemerin concentrations independently of malignancy. When logistic regression models were adjusted for age and sex, PBEF/NAMPT and OPN remained independently associated with CRC status alongside CEA, suggesting that their association with disease is not solely attributable to these demographic differences. Nevertheless, the numerical improvement in AUC over a model containing CEA, age, and sex alone did not reach statistical significance, and future studies should ideally employ age- and sex-matched control groups to minimize the influence of these confounders. Finally, although the diagnostic models combining OPN or PBEF/NAMPT with CEA showed a numerically higher AUC than CEA alone, this increase did not reach statistical significance, and the models were evaluated in the same cohort used to derive them; although bootstrap-based internal validation indicated limited overfitting, the models were not validated in an independent external cohort. Therefore, the present findings should be regarded as exploratory and confirmed in larger, multicenter studies including clinically relevant comparator groups.

Despite these limitations, our study also has several strengths. To our knowledge, this is one of the few studies evaluating three immunometabolic biomarkers simultaneously in relation to established CRC markers, together with ROC analysis and detailed evaluation according to TNM stage within a single diagnostic panel. This comprehensive approach allowed for assessment of both their individual and combined diagnostic performance. Overall, our findings suggest that OPN may complement CEA and warrants further investigation as part of a diagnostic panel for colorectal cancer; however, its clinical utility, particularly for non-invasive diagnosis, remains to be established in studies using clinically relevant comparator groups and independent validation cohorts. Larger prospective, multicenter studies are required to confirm these findings and to determine their clinical utility.

## 4. Materials and Methods

### 4.1. Patients

This was a single-center exploratory case–control study which included 76 patients diagnosed with colorectal cancer (CRC) between 2019 and 2023 (Table 5). All participants were recruited from the Department of Oncological Surgery and the Specialized Cancer Treatment Units at the Maria Skłodowska-Curie Oncology Center in Bialystok, Poland. Tumor classification and clinical staging were performed according to the Tumor–Node–Metastasis (TNM) system established by the Union for International Cancer Control (UICC).

The diagnosis of CRC was confirmed by histopathological evaluation of surgically obtained tissue specimens. Disease stage was determined based on the TNM classification, taking into account primary tumor extent (T), regional lymph node involvement (N), distant metastases (M), and histological grade (G). Before treatment initiation, each patient underwent a comprehensive diagnostic assessment including physical examination, routine laboratory investigations, and contrast-enhanced computed tomography (CT). Patients with rectal cancer additionally underwent pelvic magnetic resonance imaging (MRI). Functional status was evaluated using the Eastern Cooperative Oncology Group (ECOG) performance scale. The control cohort comprised 38 healthy volunteers without a history of malignant disease or gastrointestinal disorders. All controls had a negative colonoscopy and normal results of routine biochemical and hematological laboratory tests. The same exclusion criteria were applied to both the CRC and control groups: individuals with a body mass index (BMI) greater than 35 kg/m^2^, a previous cancer diagnosis (other than the index CRC in the case group), pregnancy, acute infection, diabetes mellitus, or chronic inflammatory, respiratory, gastrointestinal, or autoimmune disorders, including systemic lupus erythematosus and rheumatoid arthritis, were excluded from the study. Patients and controls included in this study were selected from a larger, ongoing cohort at our center, based on the availability of serum samples for measurement of PBEF/NAMPT, OPN, and chemerin. Some of the patients and controls have also contributed samples to other studies from our group evaluating different biomarker panels; CEA and CA19-9, measured routinely in all patients as part of standard clinical care, are used here, as in our previous studies, as a standard clinical comparator.

### 4.2. Biochemical Analyses

Fasting peripheral venous blood was collected in the morning from all participants before the commencement of any oncological treatment, including surgery, chemotherapy, or radiotherapy. Blood samples were drawn into serum separator tubes containing a clot activator (S-Monovette^®^, Sarstedt, Germany), centrifuged, and the obtained serum aliquots were stored at −80 °C until analysis. Serum concentrations of the investigated biomarkers were determined using a customized Human Luminex Discovery Assay (LXSAHM, R&D Systems, Abingdon, UK). Conventional tumor markers were quantified using a chemiluminescent microparticle immunoassay (CMIA) performed on an Abbott analytical platform (Abbott Laboratories, Chicago, IL, USA), in accordance with the manufacturer’s recommendations. All samples, calibration standards, and quality controls for the Luminex assay were analyzed in duplicate with an acceptance criterion of an inter-duplicate coefficient of variation below 20% to ensure analytical reliability.

### 4.3. Statistical Analysis

Statistical analyses were performed using TIBCO Statistica version 14.1.0.4 (TIBCO Software Inc., Palo Alto, CA, USA) and R version 4.5.2 (R Foundation for Statistical Computing, Vienna, Austria), with the pROC package (version 1.19.0.1) and RStudio (version 2026.08.01) used as the development environment. Data distribution was assessed using the Shapiro–Wilk test, which demonstrated that none of the analyzed variables followed a normal distribution. Consequently, non-parametric statistical methods were applied throughout the study. Comparisons between two independent groups were carried out using the Mann–Whitney U test, whereas differences among multiple groups were evaluated using the Kruskal–Wallis rank ANOVA. When an overall difference was detected, two-sided pairwise multiple comparisons of mean ranks were performed using the corresponding post hoc procedure implemented in TIBCO Statistica 14.1.0.4, with Bonferroni adjustment for multiple comparisons.

Receiver operating characteristic (ROC) curve analysis was used to evaluate the diagnostic performance of individual biomarkers and their combinations in distinguishing patients from controls. All ROC analyses, for both individual biomarkers and combined models, were performed in R using the pROC package (version 1.19.0.1), to ensure a consistent analytical pipeline. For each biomarker, the area under the curve (AUC) and its 95% confidence interval were estimated using DeLong’s method; the standard error derived from this estimate was used to test AUC against the null hypothesis of 0.5 (Wald z-test). Optimal cut-off values for individual biomarkers were selected using the Youden index (Table 4). For combined biomarker models (PBEF/NAMPT + CEA; OPN + CEA), binary logistic regression models were fitted with the respective biomarkers as independent variables and disease status as the dependent variable. The predicted probabilities generated by each logistic regression model were used as the test variable in subsequent ROC curve analysis, following the same procedure as for individual biomarkers. The optimal cut-off value for each combined model, expressed as a predicted probability threshold, was determined using the Youden index (Table 4). Sensitivity, specificity, accuracy, PPV and NPV were calculated at the corresponding optimal threshold for each biomarker and combined model, with 95% confidence intervals estimated using the Wilson method. The AUC of each combined model was formally compared with the AUC of CEA alone using a paired DeLong test, given that both curves were derived from the same subjects; results were confirmed using a bootstrap-based comparison (2000 replicates). To assess the risk of overfitting associated with evaluating combined models on the same cohort used to derive them, bootstrap-based optimism correction (500 replicates, following Harrell’s approach) was performed for each combined model: the optimism in AUC was estimated as the average difference between the AUC of a bootstrap-fitted model evaluated on the bootstrap sample and on the original sample, and this optimism was subtracted from the apparent AUC to obtain an optimism-corrected AUC. Despite this internal validation, the combined models remain exploratory and were not independently validated in an external cohort. To investigate associations between the analyzed biomarkers and clinicopathological variables, Spearman’s rank correlation coefficient was calculated. Statistical significance was defined as a *p*-value below 0.05.

## 5. Conclusions

The results of the present study indicate that, among the investigated immunometabolic biomarkers, OPN showed the strongest individual diagnostic performance, with PBEF/NAMPT showing more modest discrimination; chemerin did not show significant individual diagnostic value. The combination of OPN with CEA yielded the numerically highest AUC among all tested models. However, when formally compared with CEA alone using a paired DeLong test, this increase did not reach statistical significance, and although bootstrap-based internal validation indicated limited overfitting, the combined models were not validated in an independent external cohort. Therefore, these findings should be considered exploratory and require confirmation in independent, adequately powered cohorts using clinically relevant comparator groups. In particular, because this single-center study compares established CRC cases with healthy, colonoscopy-negative volunteers, the reported ROC findings reflect exploratory discrimination within this case–control dataset and are subject to spectrum bias; they should not be interpreted as evidence of diagnostic or screening performance in a clinically intended-use population. Nevertheless, PBEF/NAMPT and OPN showed significant stage-related changes in serum concentration, and, together with chemerin, may reflect biological processes involved in colorectal carcinogenesis that warrant further mechanistic investigation. In summary, serum OPN appears to be a promising candidate for further investigation as a complementary biomarker to CEA in colorectal cancer, although its clinical usefulness—particularly for non-invasive diagnosis—remains to be established in larger, multicenter studies with clinically relevant comparator groups before consideration for routine clinical practice.

## Figures and Tables

**Table 1:** Serum concentrations of immunometabolic biomarkers and conventional tumor markers in patients with colorectal cancer and healthy controls.

Biomarker	CRC (*n* = 76) Median (IQR) Min–Max	Controls (*n* = 38) Median (IQR) Min–Max	Nominal *p* Value *	U Value *	Rank-Biserial r (95% CI)
PBEF/NAMPT (pg/mL)	10,191.96 (7756.16–17,291.30) 3089.56–237,575.60	8179.13 (5976.25–12,770.02) 525.42–27,676.10	0.032	1087.00	0.247 (0.023–0.461)
OPN (pg/mL)	78,201.84 (42,570.52–114,204.30) 3537.98–663,582.50	34,046.86 (11,427.58–63,718.71) 1423.85–141,023.40	<0.001	730.00	0.494 (0.303–0.670)
Chemerin (pg/mL)	17,842.66 (10,458.29–25,379.10) 5851.41–49,325.30	17,040.53 (11,253.57–20,405.91) 5858.82–25,151.90	0.310	1274.00	0.118 (−0.096–0.327)
CEA (ng/mL)	2.94 (1.73–11.20) 0.50–1844.00	0.52 (0.50–1.73) 0.50–7.80	<0.001	391.50	0.729 (0.577–0.860)
CA19-9 (U/mL)	5.09 (2.42–11.90) 2.00–1357.00	3.48 (2.09–4.85) 2.00–39.30	0.022	1064.00	0.263 (0.054–0.468)

*—Mann–Whitney U test.

**Table 2:** Kruskal–Wallis and post hoc analysis of serum biomarker concentrations in healthy controls and patients with early- and advanced-stage colorectal cancer.

Parameter	PBEF/NAMPT	OPN	Chemerin	CEA	CA19-9
Kruskal–Wallis *p*-value	0.012	<0.001	<0.001	<0.001	0.013
Significant post hoc comparisons					
Control vs. Early CRC (Stage I–II)	0.011	<0.001	0.008	0.002	0.980
Control vs. Advanced CRC (Stage III–IV)	1.000	0.002	0.860	<0.001	0.011
Early CRC (Stage I–II) vs. Advanced CRC (Stage III–IV)	0.121	1.000	<0.001	<0.001	0.186

Abbreviations: CRC, colorectal cancer.

**Table 3:** Kruskal–Wallis and post hoc analysis of serum biomarker concentrations according to TNM stage.

Parameter	PBEF/NAMPT	OPN	Chemerin	CEA	CA19-9
Kruskal–Wallis *p*-value	0.009	<0.001	<0.001	<0.001	0.029
Significant post hoc comparisons					
Control vs. Stage I	0.004	0.010	0.135	0.477	1.000
Control vs. Stage II	1.000	0.009	0.164	0.002	1.000
Control vs. Stage III	1.000	0.006	1.000	<0.001	0.064
Control vs. Stage IV	1.000	0.319	0.063	<0.001	0.422
Stage I vs. Stage II	0.382	1.000	1.000	1.000	1.000
Stage I vs. Stage III	0.050	1.000	1.000	0.164	0.223
Stage I vs. Stage IV	0.265	1.000	<0.001	<0.001	0.898
Stage II vs. Stage III	1.000	1.000	1.000	1.000	1.000
Stage II vs. Stage IV	1.000	1.000	<0.001	0.046	1.000
Stage III vs. Stage IV	1.000	1.000	0.012	0.474	1.000

**Table 4:** Diagnostic performance of investigated biomarkers for colorectal cancer.

Biomarker	AUC (95% CI)	Cut-Off	*p* Value	SE (%) (95% CI)	SP (%) (95% CI)	ACC (%) (95% CI)	PPV (%) (95% CI)	NPV (%) (95% CI)
PBEF/NAMPT	0.624 (0.513–0.734)	8388.94	0.029	69.7 (58.7–78.9)	55.3 (39.7–69.9)	64.9 (55.8–73.1)	75.7 (64.5–84.2)	47.7 (33.8–62.1)
OPN	0.747 (0.655–0.839)	52,448.01	<0.001	71.1 (60.0–80.0)	68.4 (52.5–80.9)	70.2 (61.2–77.8)	81.8 (70.9–89.3)	54.2 (40.3–67.4)
Chemerin	0.559 (0.453–0.665)	25,572.57	0.275	25.0 (16.6–35.8)	100.0 (90.8–100)	50.0 (41.0–59.0)	100.0 (83.2–100)	40.0 (30.7–50.1)
CEA	0.864 (0.794–0.935)	1.43	<0.001	92.1 (83.8–96.3)	71.1 (55.2–83.0)	85.1 (77.4–90.5)	86.4 (77.3–92.2)	81.8 (65.6–91.4)
CA19-9	0.632 (0.529–0.734)	4.98	0.012	51.3 (40.3–62.2)	78.9 (63.7–88.9)	60.5 (51.4–69.0)	83.0 (69.9–91.1)	44.8 (33.5–56.6)
PBEF/NAMPT + CEA	0.875 (0.811–0.939)	0.669 ^a^	<0.001	71.1 (60.0–80.0)	89.5 (75.9–95.8)	77.2 (68.7–83.9)	93.1 (83.6–97.3)	60.7 (47.6–72.4)
OPN + CEA	0.910 (0.857–0.964)	0.626 ^a^	<0.001	82.9 (72.9–89.7)	89.5 (75.9–95.8)	85.1 (77.4–90.5)	94.0 (85.6–97.7)	72.3 (58.2–83.1)

Abbreviations: AUC, area under the ROC curve; SE, sensitivity; SP, specificity; ACC, accuracy; PPV, positive predictive value; NPV, negative predictive value. All analyses were performed in R (pROC package). AUC 95% confidence intervals were estimated using DeLong’s method; significance of AUC vs. the null value of 0.5 was assessed using a Wald z-test based on the DeLong standard error. Optimal cut-off values were determined using the Youden index, and 95% confidence intervals for SE, SP, ACC, PPV and NPV were estimated using the Wilson method. The Youden-derived cut-off values were optimized within the present cohort and should therefore be considered exploratory; their associated sensitivity and specificity require prospective validation before clinical application. PPV and NPV are sample-specific estimates reflecting the case–control composition of this study (76 CRC cases and 38 controls, corresponding to a CRC prevalence of 66.7% in this dataset) rather than the prevalence of CRC in a screening or symptomatic clinical population; these values should not be directly transported to other clinical settings without adjustment for the relevant target-population prevalence. ^a^ Cut-off expressed as predicted probability derived from a logistic regression model combining the two biomarkers.

**Table 5:** Characteristics of colorectal cancer (CRC) and healthy control groups.

Study Group		No. of Patients
Colorectal Cancer		76
	Gender:	
	Female	26
	Male	50
	Type:	
	Colon Cancer	32
	Rectal Cancer	44
	Histological subtype:	
	adenocarcinoma	68
	mucinous adenocarcinoma	7
	squamous cell carcinoma	1
	TNM Stage:	
	I	18
	II	18
	III	20
	IV	20
	Depth of tumor invasion:	
	T1	5
	T2	16
	T3	44
	T4	11
	Nodal involvement:	
	N0	41
	N1	23
	N2	12
	Distant metastasis:	
	M0	56
	M1	20
	Histological grade:	
	G1	6
	G2	61
	G3	9
	Preoperative therapy:	
	Yes	26
	No	50
	Age median (IQR)	66.5 (59.0–75.3)
	Range	32–89
Control Group		38
	Gender:	
	Female	22
	Male	16
	Age median (IQR)	48.5 (37.5–58.0)
	Range	23–75
Group comparison		*p* value
	Age (Mann–Whitney U test)	<0.001
	Sex (Fisher’s exact test)	0.026

## Data Availability

The data presented in this study are available on request from the corresponding author. Key data are stated in the text.

## Supplementary Materials

The following supporting information can be downloaded at: https://www.mdpi.com/article/10.3390/ijms27188077/s1.
