# Supplementary material for: Immunometabolic Biomarkers in Colorectal Cancer: An Exploratory Diagnostic Evaluation of PBEF/NAMPT, Chemerin, and Osteopontin
Source: Int J Mol Sci. 2026 Sep 11;27(18):8077. doi: 10.3390/ijms27188077 (PMC13606722; doi:10.3390/ijms27188077)
Supplement: Supplementary file 1 [file ijms-27-08077-s001.zip › ijms-4481127-supplementary.pdf]

Supplementary Table S1. Marker concentrations according to colorectal cancer stage.

Marker	Stage	Median	Q1	Q3
PBEF/NAMPT	I	19,956.0	13,059.1	32,592.2
	II	10,516.0	7,613.4	13,500.4
	III	9,211.5	6,441.1	11,983.9
	IV	9,538.7	8,546.2	11,049.4
OPN	I	79,594.7	57,231.6	89,991.6
	II	77,066.1	54,059.1	112,174.8
	III	84,184.1	58,810.0	116,771.1
	IV	40,398.6	23,971.6	142,263.8
Chemerin	I	20,915.0	18,162.7	27,928.0
	II	21,163.6	15,136.0	27,029.5
	III	19,478.8	10,491.8	30,526.9
	IV	9,283.9	7,837.3	11,947.4
CEA	I	1.7	1.7	2.1
	II	2.1	1.7	4.9
	III	2.8	1.7	12.1
	IV	12.4	5.1	174.9
CA19-9	I	3.5	2.1	5.3
	II	4.9	3.9	10.1
	III	7.2	4.2	29.4
	IV	8.0	2.1	38.4

Data are presented as median with first (Q1) and third (Q3) quartiles.

Supplementary Table S2. Logistic regression coefficients, odds ratios, and 95% confidence intervals for combined biomarker models

Variable	Coefficient	SE	p value	OR	95% CI (OR)
**PBEF/NAMPT + CEA (unadjusted)**					
PBEF/NAMPT	0.0001	0.00004	0.015	1.0001	1.0000–1.0002
PBEF/NAMPT (per 1000 pg/mL)	0.085	0.035	0.015	1.089	1.017–1.166
CEA	0.924	0.277	<0.001	2.52	1.58–4.63
**OPN + CEA (unadjusted)**					
OPN	0.00003	0.00001	<0.001	1.00003	1.00001–1.00005
OPN (per 1000 pg/mL)	0.028	0.008	<0.001	1.028	1.012–1.044
CEA	0.855	0.265	0.001	2.35	1.53–4.25
**PBEF/NAMPT + CEA (age- and sex-adjusted)**					
PBEF/NAMPT	0.0001	0.00005	0.023	1.0001	1.0000–1.0002
PBEF/NAMPT (per 1000 pg/mL)	0.109	0.048	0.023	1.115	1.015–1.225
CEA	0.614	0.271	0.023	1.85	1.23–3.50
Age	0.088	0.026	<0.001	1.09	1.04–1.16
Sex (male vs. female)	0.454	0.580	0.434	1.57	0.50–5.02
**OPN + CEA (age- and sex-adjusted)**					
OPN	0.00003	0.00001	0.002	1.00003	1.00001–1.00005
OPN (per 1000 pg/mL)	0.029	0.009	0.002	1.029	1.011–1.049
CEA	0.617	0.234	0.009	1.85	1.28–3.31
Age	0.085	0.025	0.001	1.09	1.04–1.15
Sex (male vs. female)	0.371	0.633	0.558	1.45	0.41–5.11

Coefficient, Regression coefficient (log-odds) from binary logistic regression; SE, standard error; OR, odds ratio; CI, confidence interval (profile likelihood for per-1 pg/mL estimates; Wald-based for per-1000 pg/mL estimates). Unadjusted models include the indicated biomarker and CEA as predictors of CRC status. Age- and sex-adjusted models additionally include age (years) and sex (male vs. female) as covariates. Because PBEF/NAMPT and OPN are measured in absolute concentration units (pg/mL) spanning a wide range, odds ratios per 1 pg/mL increase are close to 1.0; odds ratios per 1000 pg/mL increase are additionally reported for clinical interpretability. Some models showed evidence of quasi-complete separation, a common occurrence when several correlated, individually strong predictors (e.g., OPN, CEA, and age) are combined in a model of moderate sample size.

Supplementary Table S3. Diagnostic performance for early colorectal cancer (Stage I+II) vs. controls (n = 36 vs. n = 38).

Marker	AUC (95% CI)	Cut-off	p value	SE (%) (95% CI)	SP (%) (95% CI)	ACC (%) (95% CI)	PPV (%) (95% CI)	NPV (%) (95% CI)
PBEF/NAMPT	0.686 (0.564–0.808)	9755.95	0.003	69.4 (53.1–82.0)	60.5 (44.7–74.4)	64.9 (53.5–74.8)	62.5 (47.0–75.8)	67.6 (50.8–80.9)
OPN	0.795 (0.693–0.896)	52448.01	<0.001	80.6 (65.0–90.2)	68.4 (52.5–80.9)	74.3 (63.3–82.9)	70.7 (55.5–82.4)	78.8 (62.2–89.3)
CEA	0.788 (0.683–0.893)	1.43	<0.001	83.3 (68.1–92.1)	71.1 (55.2–83.0)	77.0 (66.3–85.1)	73.2 (58.1–84.3)	81.8 (65.6–91.4)
PBEF/NAMPT + CEA	0.829 (0.734–0.924)	0.550[a]	<0.001	66.7 (50.3–79.8)	89.5 (75.9–95.8)	78.4 (67.7–86.2)	85.7 (68.5–94.3)	73.9 (59.7–84.4)
OPN + CEA	0.882 (0.805–0.958)	0.407[a]	<0.001	88.9 (74.7–95.6)	73.7 (58.0–85.0)	81.1 (70.7–88.4)	76.2 (61.5–86.5)	87.5 (71.9–95.0)

[a] Cut-off expressed as predicted probability from the logistic regression model.
